# *Sasa quelpaertensis* Leaf Extract Inhibits Colon Cancer by Regulating Cancer Cell Stemness *in Vitro* and *in Vivo*

**DOI:** 10.3390/ijms16059976

**Published:** 2015-04-30

**Authors:** Soo Jin Min, Ji Ye Lim, Haeng Ran Kim, Se-Jae Kim, Yuri Kim

**Affiliations:** 1 Department of Nutritional Science and Food Management, Ewha Womans University, Seoul 120-750, Korea; E-Mails: 009267@naver.com (S.J.M.); godlovesme86@hanmail.net (J.Y.L.); 2 National Academy of Agricultural Science, Rural Development Administration, Jeollabuk-do 565-851, Korea; E-Mail: kimhrr@korea.kr; 3 Department of Biology, Jeju National University, Jejusi, Jeju 690-756, Korea; E-Mail: jejusasa@naver.com

**Keywords:** *Sasa quelpaertensis* Nakai leaf extract, colon cancer, cancer stem cells, self-renewal, differentiation

## Abstract

A rare subpopulation of cancer cells, termed cancer stem cells (CSCs), may be responsible for tumor relapse and resistance to conventional chemotherapy. The development of a non-toxic, natural treatment for the elimination of CSCs is considered a strategy for cancer treatment with minimal side effects. In the present study, the potential for *Sasa quelpaertensis* leaf extract (SQE) and its two bioactive compounds, tricin and *p*-coumaric acid, to exert anti-CSC effects by suppressing cancer stemness characteristics were evaluated in colon cancer cells. CD133^+^CD44^+^ cells were isolated from HT29 and HCT116 cell lines using flow-activated cell sorting (FACs). SQE treatment was found to significantly suppress the self-renewal capacity of both cell lines. SQE treatment was also associated with the down-regulation of β-catenin and phosphorylated GSK3β, while significantly enhancing cell differentiation by up-regulating CK20 expression and blocking the expression of several stem cell markers, including DLK1, Notch1, and Sox-2. *In vivo*, SQE supplementation suppressed tumor growth in a xenograft model by down-regulating stem cell markers and β-catenin as well as HIF-1α signaling. Compared with two bioactive compounds of SQE, SQE exhibited the most effective anti-CSC properties. Taken together, these results provide evidence that SQE inhibits colon cancer by regulating the characteristics of CSCs.

## 1. Introduction

Worldwide, colon cancer is one of the most common types of cancer diagnosed, and it is a major cause of cancer-related morbidity and mortality in both men and women. Colon cancer is responsible for 1.4 million new cases and nearly 700,000 deaths worldwide [1]. Despite advances in the screening used for colon cancer and the emergence of new targeted therapeutic combinations, nearly 50% of colon cancer patients experience recurrence. Thus, it is hypothesized that colon cancer is driven by a rare-subpopulation of self-renewing cancer cells, termed cancer stem cells (CSCs) [2]. Accordingly, failure to eliminate CSCs may be critical for metastasis and cancer relapse following therapeutic treatment [3]. Therefore, targeting of CSCs may represent a key therapeutic strategy for the complete treatment of diseases that are maintained by these CSC populations.

In most cases, CSCs have been identified based on their expression of specific cell surface markers, including CD133, CD44, and aldehyde dehydrogenase (ALDH1). CD133 (also known as prominin-1) is a type I transmembrane glycoprotein that has been characterized as a cell surface marker of CSCs [4]. O’Brian and colleague were the first to demonstrate that only a small subset of CSCs isolated from a CD133^+^ population were capable of growing as clonospheres in serum-free sphere media, and these cells could initiate tumor growth in a serial xenograft mouse model [5]. CD133^+^ cells have also been found to maintain long-term expression of CD133 when grown in sphere media [6]. CD44 is a hyaluronan receptor that plays a critical role in the homing and colonization of adult stem cells, CSCs, and metastasizing cancer cells [7]. Similarly, single CD44^+^ colon cancer cells have been shown to form spheres in serum-free sphere media and have been used to establish xenograft tumor models *in vivo* [8]. Consequently, CD44 has been reported to be a marker for colon CSCs. While colon cancer cells express both CD133 and CD44, the presence of these markers alone is probably insufficient to identify CSCs [9]. Furthermore, cells expressing CD133^+^ and CD44^+^ have exhibited greater tumorigenicity than cells expressing either marker alone [10]. Taken together, these results suggest that a combination of markers are needed to identify the CSC population in human colon cancer cells.

CSCs have the capacity to undergo pluripotent differentiation, self-renewal, and tumorigenicity, and these can lead to resistance to chemotherapy [11,12]. Induction of terminal differentiation to inhibit self-renewal may represent a valid treatment option for eliminating CSCs. A number of stem cell markers are expressed by CSCs. Of these, Drosophila delta-like 1 homologue (DLK1) is a member of the epidermal growth factor-like homeotic protein family and has been reported to regulate the differentiation of adipocytes, hematopoietic stem cells, and neuronal and hepatic CSCs [13,14]. SRY-related HMG-box-2 (*Sox-2*) is a gene that plays a role in the maintenance of a progenitor state and its transcription has been detected in all of the tumors tested to date [15]. Another stem cell marker, Notch homolog 1 (Notch1), has a fundamental role in regulating proliferation of CSCs [16].

Wnt/β-catenin signaling is one of the key pathways involved in the transformation of normal colonic epithelial cells into colon cancer cells and self-renewing CSCs [17,18]. Briefly, translocation of β-catenin into the nucleus leads to its binding of T cell factor/lymphoid enhancer factor (TCF/LEF) and the activation of Wnt target genes [18]. Glycogen synthase kinase-3β (GSK3β) phosphorylates β-catenin to regulate its levels of expression via ubiquitination and targeted degradation [19]. Hypoxia further increases the clonogenicity of tumor cells and the intrinsic tumorigenic potential of tumor cells by arresting tumorigenic cells in their undifferentiated state and maintaining their stem cell potential [20]. Hypoxia-inducible factor-1 (HIF-1) is a critical regulator of the tumor cell response to hypoxia, and it consists of an oxygen-dependent α-subunit (HIF-1α) and an oxygen-independent β-subunit (HIF-1β). Upon stabilization of the α-subunit, HIF-1α translocates to the nucleus and activates various genes, including vascular endothelial growth factor (VEGF) [21]. Thus, regulation of CSCs and hypoxic cancer cells may represent a more efficient therapeutic strategy for the targeting of highly resistant cancers.

*Sasa* (poaceae), known as bamboo grass, is widely grown in Asian countries, including Korea, China, and Japan [22]. *Sasa* leaves are commonly considered to be beneficial for diabetes, obesity, ulcers, inflammation, and cancer [23,24,25]. Previously, various *Sasa* species and their bioactive compounds have been shown to exhibit anti-cancer and anti-tumor properties [24,26,27]. For example, *Sasa kumaizawa* extract has been shown to mediate immunopotentiating and cancer preventive effects in a 7,12-dimethylbenz[α]anthracene (DMBA)-induced rat tumor model [28]. In SHN mice, an anti-mammary tumor effect following treatment with an alkaline extract of *Sasa senanensis* Rehder (also known as Sasa Health) was observed [29]. *Sasa quelpaertensis* Nakai is native to Korea and is only grown on Mt. Halla (Jeju Island, Korea) [30]. *Sasa quelpaertensis* leaves contain a mixture of polysaccharides and polyphenols, including *p*-coumaric acid and tricin, which may mediate the anticancer effects observed for *Sasa quelpaertensis* extracts (SQE) [25,31]*.* Byun *et al.* [32] have recently reported an pro-apoptotic effect for *Sasa quelpaertensis* Nakai on HT29 colon cancer cells, while an anti-cancer effect was observed following the treatment of lung cancer cells with a combination of *Sasa quelpaertensis* Nakai leaf extract and cisplatin [30]. However, there is little known about the role of SQE and its bioactive compounds in mediating or inducing the differentiation, self-renewal capacity, and tumorigenicity of colon CSCs. Therefore, the aim of the present study was to investigate the effects of SQE on characteristics of colon CSCs.

## 2. Results and Discussion

### 2.1. Isolation of CD133^+^CD44^+^ HT29 and CD133^+^CD44^+^ HCT116 Cells by FACS (Flow-Activated Cell Sorting)

**Figure 1:**
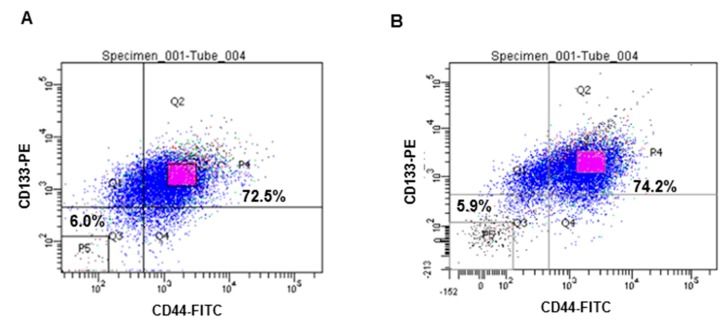
Isolation of CD133^+^CD44^+^ HT29 and CD133^+^CD44^+^ HCT116 cells by FACS*.* Expression of the CSCs markers, CD133 and CD44, was calculated using flow cytometry (**A**) HT 29 cells; (**B**) HCT 116 cells.

Expression of the CSC markers, CD133 and CD44, were analyzed by FACS. CD133^+^CD44^+^ double-stained cells were isolated from both HT29 and HCT116 cell lines and this subpopulation was more than 70% for each cell line. According to their fluorescence intensity values, , the top 20% of the isolated cells with the highest fluorescence intensities of the isolated cells were designated CD133^+^CD44^+^ CSCs, while 20% of the cells with the lowest fluorescence intensities of the isolated cells were used as control cells for further experiments (Figure 1A,B).

### 2.2. Effects of SQE (*Sasa quelpaertensis* Leaf Extract), p-Coumaric Acid, and Tricin on the Self-Renewal Characteristics of Colon CSCs (Cancer Stem Cells)

**Figure 2:**
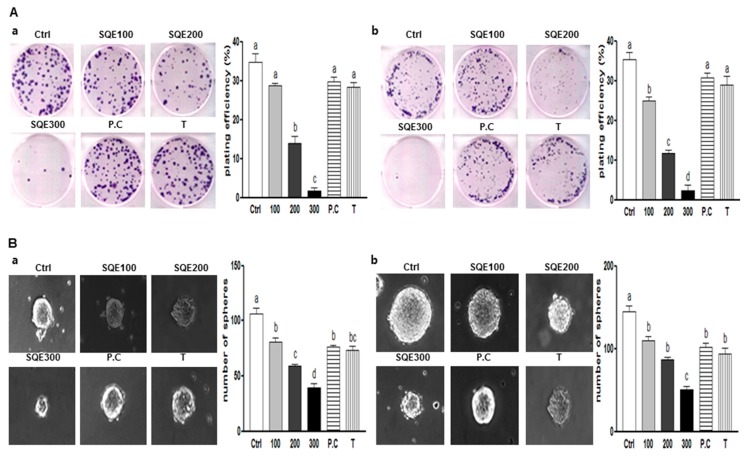
Effects of SQE, *p*-coumaric acid, and tricin on self-renewal characteristics of colon CSCs. CD133^+^CD44^+^ HT29 cells (**a**) and CD133^+^CD44^+^ HCT116 cells (**b**) were treated with SQE (0, 100, 200, or 300 μg/mL), or *p*-coumaric acid (1.8 μM) and tricin (0.7 μM) at concentrations equivalent to that contained in 300 μg/mL SQE. (**A**) After eight days, the resulting colonies were fixed and stained. Microscopy images of colony formation were obtained (magnification, 100×, **left** panel) and the number of colonies was recorded (**right** panel). Plating efficiency (%) = (number of colonies)/(total cell number) × 100; (**B**) Sphere formation was analyzed for both cell lines and images were obtained with phase contrast microscopy (**left** panel, magnification, 100×). The number of spheres was recorded (**right** panel). The letter labels on the histogram indicate the values that significantly differed from each other (*p* < 0.05) according to one-way ANOVA for multiple comparisons. Ctrl, Control; SQE, *Sasa quelpaertensis* extract; P.C, *p-*coumaric acid; T, tricin.

One of the significant characteristics of CSCs is the capacity for self-renewal. Clonogenic assays are used to analyze the ability of a single cell to form a colony, a process which requires reproductive integrity and represents the self-renewal potential of CSCs [14]. When HT29 cells were treated with various concentrations of SQE (0, 100, 200, or 300 μg/mL), as well as *p*-coumaric acid and tricin at concentrations equivalent to 300 μg/mL SQE, for eight days, clonogenic formation was suppressed in the presence of 200 and 300 μg/mL SQE (59.6% and 95.2% compared to control cells; *p* < 0.001 and *p* < 0.001, respectively). *p*-Coumaric acid and tricin also tended to suppress colony formation, although the differences were not statistically significant (Figure 2Aa). For the HCT116 cells, SQE treatment inhibited colony formation in a dose-dependent manner (*p* < 0.01), while *p*-coumaric acid and tricin only weakly inhibited colony formation (Figure 2Ab).

Another widely used assay to analyze the self-renewal capacity of CSCs is the sphere formation assay. In this assay, the ability of cells to grow as non-adherent spheroids in serum-free CSC medium is examined [33]. In the present study, treatment with SQE, *p*-coumaric acid, and tricin resulted in a marked decrease in the size and number of the spheres that formed compared with untreated HT29 and HCT 116 cells (Figure 2B). The greatest disruption of sphere formation was observed in the presence of 300 μg/mL SQE, and this resulted in a 62.9% and 65.1% reduction in sphere formation in HT29 cells and HCT 116 cells, respectively (*p* < 0.001 and *p* < 0.001, respectively). Taken together, these results suggest that SQE was more effective in suppressing the self-renewal capacity of colon CSCs than *p*-coumaric acid or tricin alone.

### 2.3. Effects of SQE, p-Coumaric Acid, and Tricin on Wnt/β-Catenin Signaling

**Figure 3:**
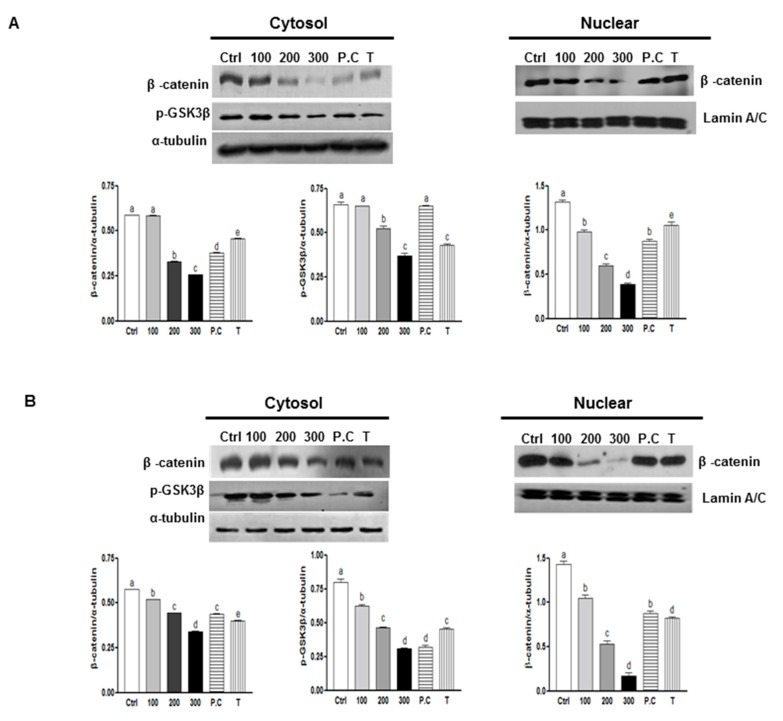
Effects of SQE, p-coumaric acid, and tricin on Wnt/β-catenin signaling. Expression levels of β-catenin and *p*-GSK3β were examined using Western blot assays in HT 29 cells (**A**) and HCT 116 cells (**B**), and detection of α-tubulin and Lamin A/C were used as loading controls. The letter labels on the histogram indicate the values that significantly differed from each other (*p* < 0.05) according to one-way ANOVA for multiple comparisons. Ctrl, Control; SQE, *Sasa quelpaertensis* extract; P.C, *p-*coumaric acid; T, tricin.

The Wnt/β-catenin signaling pathway is critical for promoting the self-renewal capacity of CSCs and it helps maintain normal colon stem cells [34]. To investigate the mechanisms that may contribute to the effect of SQE on the self-renewal capacity of colon CSCs, expression of β-catenin was examined. As shown in Figure 3, SQE treatment down-regulated expression of β-catenin in both cell lines. In particular, 300 μg/mL SQE significantly down-regulated levels of β-catenin by up to 55.9% in HT29 cells and by 40.8% in HCT116 cells compared to control cells (Figure 3; *p* < 0.001 and *p* < 0.001, respectively). *p*-Coumaric acid and tricin also significantly down-regulated both cytosolic and nuclear β-catenin expression, albeit less effectively than SQE (*p* < 0.001 and *p* < 0.001, respectively). 

GSK3β modulates levels of intracellular β-catenin by promoting ubiquitin-targeted degradation of β-catenin [35]. Phosphorylation of GSK3β at Ser9 leads to a decreased activity of this enzyme, which leads to stabilized levels of β-catenin [36]. In the present study, a decreased in phosphorylation of GSK3β from 43.8% and 62.2% was detected in cells from both cell lines that were treated with 300 μg/mL SQE. Tricin treatment also decreased the levels of GSK3β phosphorylation (Figure 3). Taken together, these results indicate that SQE is highly effective in decreasing the self-renewal capacity of colon CSCs by inactivating GSK3β phosphorylation at Ser9, thereby leading to β-catenin degradation.

### 2.4. Effects of SQE, p-Coumaric Acid, and Tricin on Cell Differentiation in Colon CSCs

**Figure 4:**
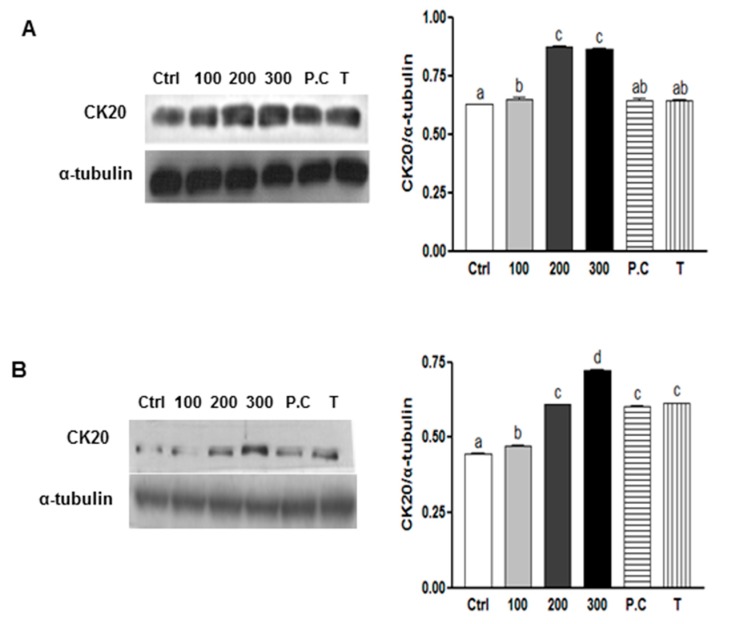
Effects of SQE, *p*-coumaric acid, and tricin on cell differentiation in colon CSCs. CD133^+^CD44^+^ HT29 cells (**A**) and CD133^+^CD44^+^ HCT116 cells (**B**) were treated with SQE (0, 100, 200, or 300 μg/mL), or *p*-coumaric acid (1.8 μM) and tricin (0.7 μM) at concentrations equivalent to that contained in 300 μg/mL SQE. Levels of protein expressions for Cytokeratin 20 (CK20) were examined by Western blot analysis, and detection of α-tubulin was used as a loading control. The letter labels on the histogram indicate the values that significantly differed from each other (*p* < 0.05) according to one-way ANOVA for multiple comparisons. Ctrl, Control; SQE, *Sasa quelpaertensis* extract; P.C, *p-*coumaric acid; T, tricin.

Another important characteristic of CSCs is their capability to undergo differentiation [3]. To evaluate the capacity for SQE, *p*-coumaric acid, and tricin to induce cell differentiation, expression of CK20, a differentiation marker of colon CSCs [37], was examined in CD133^+^CD44^+^ cells isolated from HT29 and HCT116 cell lines. The effect of SQE on the differentiation of colon CSCs was also examined by treating cells with various concentrations of SQE, *p*-coumaric acid, and tricin for eight days. In both cell lines, treatment with SQE resulted in an increase in expression levels of CK20 (Figure 4). However, treatment with *p*-coumaric acid and tricin only increased expression levels of CK20 in the HT29 cells (Figure 4A).

### 2.5. Effects of SQE, p-Coumaric Acid, and Tricin on the Expression of Stem Cell Markers and VEGF (Vascular Endothelial Growth Factor) in Colon CSCs

**Figure 5:**
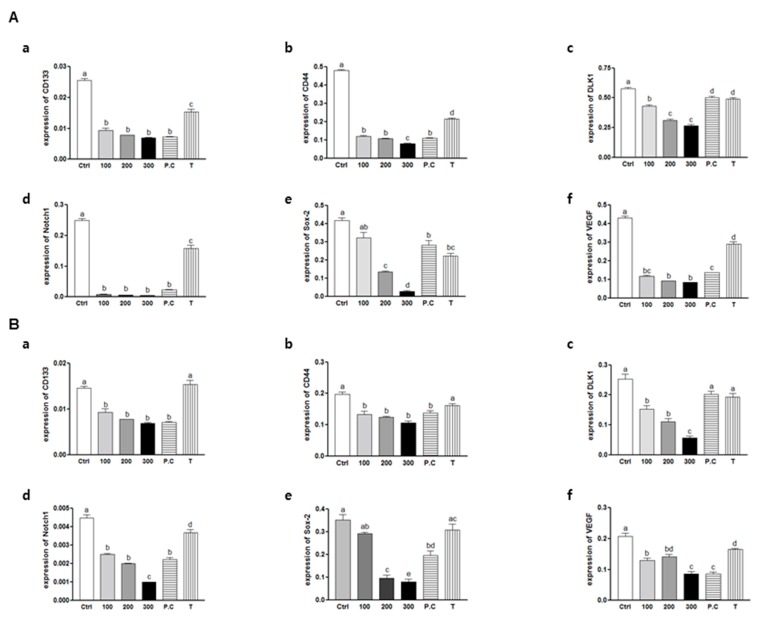
Effects of SQE, *p*-coumaric acid, and tricin on mRNA levels of CD133, CD44, DLK1, Notch1, Sox-2, and VEGF in colon CSCs. CD133^+^CD44^+^ HT29 cells (**A**) and CD133^+^CD44^+^ HCT116 cells (**B**) were treated with SQE (0, 100, 200, or 300 μg/mL), or *p*-coumaric acid (1.8 μM) and tricin (0.7 μM) at concentrations equivalent to that contained in 300 μg/mL SQE. Levels of mRNA for (**a**) *CD133*; (**b**) *CD44*; (**c**) *DLK1*; (**d**) *Notch1*; (**e**) *Sox-2*; and (**f**) *VEGF* were examined using real-time PCR, with detection of β-actin used as an internal control. The letter labels on the histogram indicate the values that significantly differed from each other (*p* < 0.05) according to one-way ANOVA for multiple comparisons. Ctrl, Control; SQE, *Sasa quelpaertensis* extract; P.C, *p-*coumaric acid; T, tricin.

The primary objective of the current study was to determine whether SQE can eliminate colon CSCs. To accomplish this objective, mRNA levels of stem cell markers, including *CD133*, *CD44*, *DLK1*, *Notch1*, *Sox-2*, and *VEGF* (a potent growth factor for blood vessel endothelial cells [38]) were assayed using real-time PCR. The expressions of all six markers were significantly down-regulated at all doses of SQE, *p*-coumaric acid, and tricin in both CD133^+^CD44^+^ HT29 and HCT116 cells (Figure 5). These results suggest that SQE and its bioactive compounds, *p*-coumaric acid and tricin, are highly effective in suppressing CSC markers and in eliminating CD133^+^CD44^+^ HT29 and HCT116 cells.

**Figure 6:**
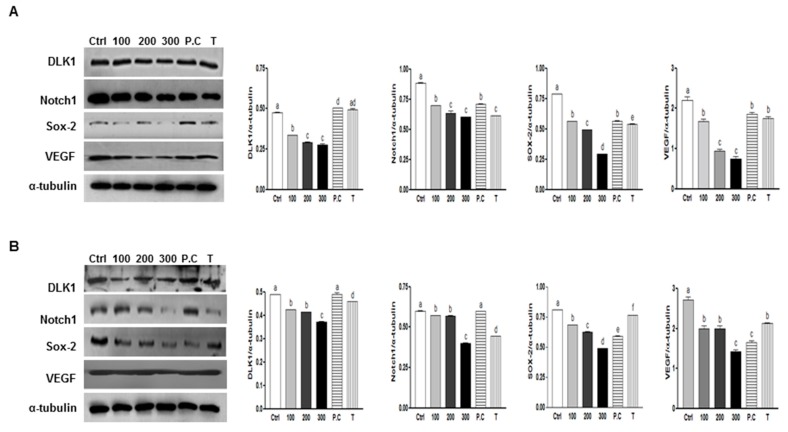
Effects of SQE, *p*-coumaric acid, and tricin on the protein expression of stem cell markers and VEGF in colon CSCs. CD133^+^CD44^+^ HT29 cells (**A**) and CD133^+^CD44^+^ HCT116 cells (**B**) were treated with SQE (0, 100, 200, or 300 μg/mL), or *p*-coumaric acid (1.8 μM) and tricin (0.7 μM) at concentrations equivalent to that contained in 300 μg/mL SQE. Levels of protein expressions for DLK1, Notch1, Sox-2, and VEGF were examined by Western blot analysis, and detection of α-tubulin was used as a loading control. The letter labels on the histogram indicate the values that significantly differed from each other (*p* < 0.05) according to one-way ANOVA for multiple comparisons. Ctrl, Control; SQE, *Sasa quelpaertensis* extract; P.C, *p-*coumaric acid; T, tricin.

To investigate whether SQE, *p*-coumaric acid, and tricin suppress protein expression levels of several well-known CSC markers were assayed, including DLK1, Notch 1, and Sox-2 and VEGF following treatment with SQE, *p*-coumaric acid, or tricin. Treatment with SQE down-regulated expression levels of these markers in both CD133^+^CD44^+^ cells isolated from the HT29 and HCT116 cells (Figure 6). In comparison, *p*-coumaric acid and tricin were less effective at down-regulating the stem cell markers assayed compared with SQE.

### 2.6. Effect of Combination of p-Coumaric Acid and Tricin on Clonogenicity and Stem Cell Marker, Notch1

**Figure 7:**
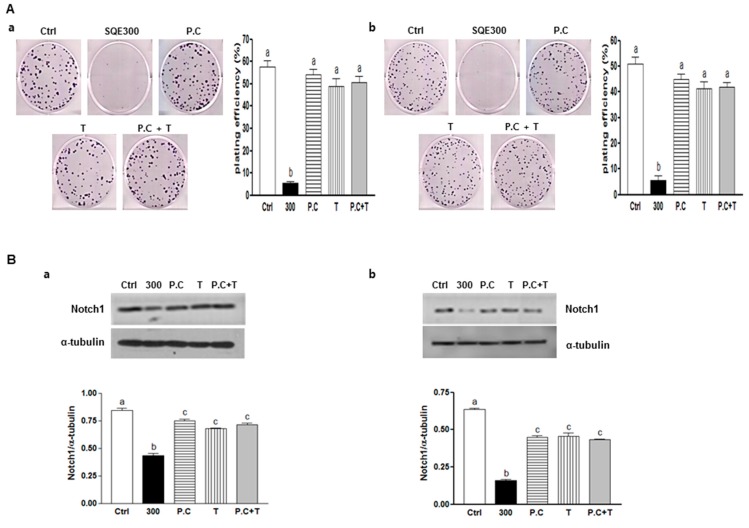
Effect of combination of *p*-coumaric acid and tricin on clonogenicity and stem cell marker, Notch1. CD133^+^CD44^+^ HT29 cells (**a**) and CD133^+^CD44^+ ^HCT116 cells (**b**) were treated with SQE (300 μg/mL), *p*-coumaric acid (1.8 μM)*,* tricin (0.7 μM), or combination of *p*-coumaric acid (1.8 μM) and tricin (0.7 μM). (**A**) After eight days, the resulting colonies were fixed and stained. Microscopy images of colony formation were obtained (magnification, 100×, left panel) and the number of colonies was recorded (right panel). Plating efficiency (%) = (number of colonies)/(total cell number) × 100; (**B**) Levels of protein expressions for Notch1 was examined by Western blot analysis, and detection of α-tubulin was used as a loading control. The letter labels on the histogram indicate the values that significantly differed from each other (*p* < 0.05) according to one-way ANOVA for multiple comparisons. Ctrl, Control; SQE, *Sasa quelpaertensis* extract; P.C, *p-*coumaric acid; T, tricin.

To investigate the synergism or additive effect between *p*-coumaric acid and tricin in SQE, the combination of *p*-coumaric acid and tricin was treated and then clonogenic capacity and level of one of stem cell markers, Notch 1 was analyzed (Figure 7). Clonogenic formation and Notch 1 expression was suppressed in the presence of 300 μg/mL SQE (89% compared to control cells; *p* < 0.001, respectively). However, combination of *p*-coumaric acid and tricin induced less of an effect compared to SQE or each of the compounds alone. Therefore, these findings confirm that other components of SQE are necessary for the anti-CSC effects of SQE.

### 2.7. Effect of SQE on Tumorigenicity and CSC Marker Expression of CD133^+^CD44^+^ HT29 Cells in Vivo

**Figure 8:**
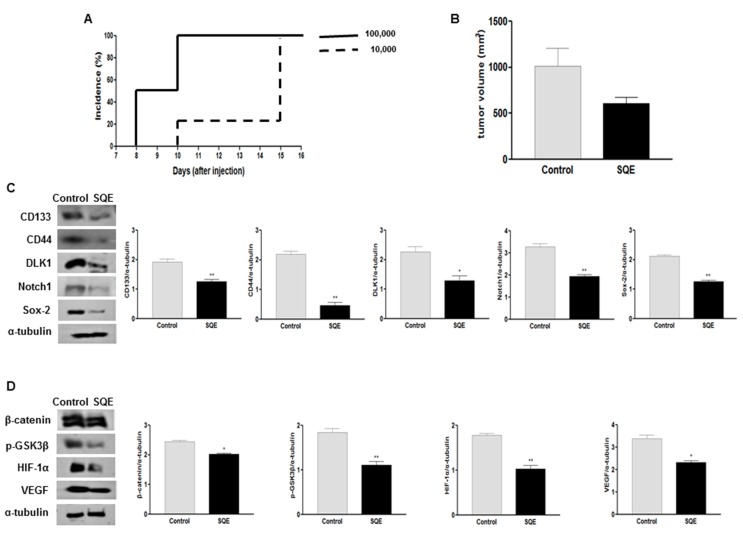
Effect of SQE on tumorigenicity and CSC marker expression of CD133^+^CD44^+^ HT29 cells *in vivo*. (**A**) The tumorigenicity of CD133^+^CD44^+^ double-sorted cells (10,000 or 100,000 cells) was examined following the subcutaneous injection of these cells into nude mice; (**B**) Tumor volume was measured and compared between the control group and the SQE (300 mg/kg body weight) supplemented group; (**C**,**D**) Expression levels of various CSC markers, including CD133, CD44, DLK1, Notch1, and Sox-2 (**C**), as well as self-renewal and metastasis signaling-related markers, including β-catenin, *p*-GSK3β, HIF-1α, and VEGF (**D**), were detected by Western blot analysis. Detection of α-tubulin was used as a loading control. Representative figures are shown (left panel). Quantification of each protein level was shown with a bar graph. Bar represented the mean ± SEM. * and ** show significantly difference between two groups by Student’s *t*-test (*p* < 0.05). SQE, *Sasa quelpaertensis* leaf extract.

In the present study, SQE treatment was found to inhibit cancer cell stemness *in vitro* by increasing the expression of differentiation markers and decreasing the expression of CSC markers. When CD133^+^CD44^+^ HT29 cells were subcutaneously injected into nude mice, as few as 1 × 10^4^ sorted cells were capable of establishing a tumor within 2 weeks (Figure 8A), thereby demonstrating the CSC potential of these cells. To investigate the anti-tumorigenic effect of SQE supplementation *in vivo*, CD133^+^CD44^+^ HT29 cells (1 × 10^4^/animal) were injected into nude mice. At the time of sacrifice, tumor incidence between the mice that received SQE supplementation and the control mice did not differ (data not shown). However, the mean tumor volume for the SQE supplemented group was 609.8 ± 226.8 mm^3^ compared with 1016.7 ± 722.1 mm^3^ for the Control group (Figure 8B). This represented a 40.1% decrease in tumor volume for the SQE treated mice, although this difference was not statistically significant. These data suggest that SQE supplementation weakly inhibits tumor growth *in vivo*.

To confirm these results, the expression of several CSC markers, including CD133, CD44, DLK1, Notch1, and Sox-2 were analyzed by Western blot. Significantly lower levels of expression were detected for all five targets in the SQE samples (Figure 8C). In addition, SQE samples exhibited 17.1% lower levels of β-catenin (*p* = 0.01) and 39.5% lower levels of phosphorylation of GSK3β at Ser9 (*p* < 0.004) compared with the Control group (Figure 8D).

Hypoxic tumors appear to be highly tumorigenic, poorly differentiated, and they express certain CSC markers [39,40]. Expression of HIF-1α is increased in poorly differentiated cancers and this leads to activation of the HIF-1α targeted gene, *VEGF* [21,41]. In the present study, expression levels of HIF-1α and VEGF were 42.2% and 31.5% lower in the SQE supplemented group compared with the Control group, respectively (*p* = 0.0012 and *p* = 0.0036, respectively) (Figure 8D). Taken together, these results suggest that SQE suppresses colon cancer tumor growth by suppressing the stemness of the CSC population present and by regulating levels of HIF-1α and its downstream target gene, *VEGF*.

### 2.8. Discussion

Despite the availability of surgical resection, radiation, and chemotherapy for the treatment of colon cancer, nearly 50% of patients develop resistance to treatment and experience tumor relapse or metastasis [42,43]. Malignancies originate from a small subset of cancer cells, termed CSCs, and these are capable of initiating and maintaining tumor growth, as well as promoting cell invasion and drug resistance [44]. Thus, the successful elimination of CSCs represents an effective strategy for achieving complete remission of colon cancers, and the identification of CSCs is a key first step.

There are several colon CSC markers, including CD133, CD44, CD166, CD24, and aldehyde dehydrogenase 1 (ALDH1) [45]. Among these, CD133 was the first identified as a cancer-initiating marker or CSC marker when a CD133^+^ subpopulation was found to be tumorigenic in a serial xenograft assay in nude mice [5]. Clinically, higher levels of CD133 expression have been associated with a poor prognosis for advanced colon cancer patients, and this has supported the significance of CD133 expression in colon cancer [46]. CD133^+^ cell populations from tumors have also been found to express CD44. The stem-like epithelial specific CD44^+^ cells indicates that CD44 represents another marker of colon CSCs [8]. In the present study, when CD133^+^ or CD44^+^ cells were labeled, they were found to be more than 90% of total cells in both cell lines (data not shown), and more than 70% of cells were CD133^+^CD44^+^ double-labeled in these cell lines. When these double-positive cells were injected subcutaneously into nude mice (1.0 × 10^4^ cells/mouse), a model which has provided a reliable and sensitive *in vivo* system for the study of human colon CSCs, 100% of the mice developed tumors. Similarly, in a previous study, only the CD133^+^CD44^+^ population exhibited tumorigenicity compared to the CD133^−^CD44^−^, CD133^+^CD44^−^, and CD133^−^CD44^+^ cells [10]. These results confirmed that the use of multiple markers to identify CSCs is more reliable than the use of only a single marker.

*Sasa senanensis* leaf extracts, which contain a higher level of phenolics have exhibited immunostimulation-mediated antitumor activity [47], while the antiproliferative and apoptotic effects of *Sasa quelpaertensis* Nakai have been reported in six human cancer cell lines, including A549, MCF-7, HeG-2, Hela, HCT116, and A375 [48]. Recently, it has also been reported that SQE alone, or in combination with cisplatin exerts anti-CSC and antimetastatic effects in H1299 and A549 human lung cancer cells by inhibiting the phosphorylation of phosphoinositide-3-kinase (PI3K) and by activating mammalian target of rapamycin (mTOR) [30]. To the best our knowledge, the present study is the first to demonstrate that SQE mediates an anti-colon cancer stemness effect by suppressing self-renewal, increasing cell differentiation, and inhibiting cell tumorigenicity of the colon CSCs.

In the present study, *in vitro* clonogenic and sphere formation assays were performed and analyzed the inhibitory effect of SQE on the self-renewal capacity of colon CSCs. The ability of CSCs to form colonies from a single cell, and to maintain anchorage-independent sphere formation in serum-free medium, has been shown to be directly proportional to the number of self-renewal cells present [3]. In the present study, CD133^+^CD44^+^ cells isolated from both HT29 and HCT116 cell lines were able to form colonies and spheroids. However, in the presence of SQE, the subsequent formation of colonies and spheres was suppressed. The Wnt/β-catenin signaling pathway is critical for promoting the self-renewal capacity of CSCs [18], and aberrant Wnt/β-catenin signaling is an early event in most human colorectal cancers [34]. It has also been reported that β-catenin localized to the nucleus in cells expressing CD133 and CD44 [8]. In the present study SQE down-regulated expression of β-catenin and decreased phosphorylation of GSK3β. Both of these results, support a SQE-mediated effect on colon CSC stemness that is induced via potent inhibitory effect on Wnt/β-catenin signaling. Other natural dietary compounds, including curcumin and turmeric have also been shown to attenuate the self-renewal capacity of colon cancer cells via β-catenin signaling through to Wnt-3a [49]. Similarly, sulforaphane derived from broccoli has been reported to regulate the Wnt/β-catenin self-renewal pathway [50].

Furthermore, SQE treatment enhanced the differentiation of CD133^+^CD44^+^ cells by upregulating the colonic epithelial differentiation marker, CK20. In addition, HCT116 cells have been characterized as not very differentiated and a highly aggressive cell line. In contrast, HT29 cells appear to possess some capacity for differentiating into enterocytes and mucin-expressing cell types [51]. However, for both cell lines in the present study, SQE treatment resulted in the up-regulation of the differentiation marker, CK20. Not many studies have shown the efficacy of naturally occurring dietary compounds on the differentiation of colon CSCs. Omega-3 eicosapentaenoic acid (EPA) has been shown to decrease levels of CD133^+^ colon CSCs by upregulating CK20 [52], and a carotenoid present in orange colored vegetables, β-carotene, has also been shown to induce the neuronal differentiation of human neuroblastoma cells [53]. Highly expressed stem cell markers, including DLK1, Notch1, and Sox-2, are also important for stem cell characteristics. In the present study, the downregulation of these CSC markers by SQE was associated with the suppression of CSC maintenance. In the present study, expressions levels of DLK1, Notch1, and Sox-2 were significantly down-regulated following treatment with SQE both *in vitro* and *in vivo*.

*In vivo*, SQE supplementation (300 mg/kg b.w.) was found to weakly inhibit colon tumor growth, yet it significantly suppressed the expression of CSC markers, Wnt/β-catenin signaling, and HIF-1α signaling in the tumors that formed. Previously, administration of SQE at 300 mg/kg b.w. every day for seven weeks suppressed dextran sulfate sodium (DSS)-induced colitis in a mouse model [54]. For patients with inflammatory bowel disease, such as ulceritive colitis, the chronic inflammatory state of these patients is considered to be a contributing factor to the development of colorectal cancer [55]. Based on the present results, it is possible that treatment with 300 mg/kg b.w. SQE may prevent chronic inflammation in the early stages of colon cancer, yet may not be sufficient to inhibit tumor growth, especially the growth of more malignant CSCs. Thus, it is possible that a higher dose may be required for the latter compared with the former. Further clinical study is warranted to confirm the present results.

Other bio-active compounds have been derived from leaves of the *Sasa* species. For example, lignin and polysaccharides from the *Sasa* species have been reported to exhibit anti-tumor activity [56], and Sasa Health extracts containing polysaccharides, cholorophyllin, lignin, and flavonoids have exhibited an anti-tumor effect in Her2/NeuN mice [24]. Many phytochemicals mediate strong antioxidant activity and anti-inflammatory effects by suppressing either the initiation or promotion step of carcinogenesis [57]. SQE contains various polyphenols, including *p*-coumaric acid and tricin, which regulate the metabolic activation of potential carcinogens and are recognized by xenobiotic metabolizing enzymes [25,58]. Previously, it was reported that *p*-coumaric acid and tricin, bio-active compounds in SQE, exerted anti-inflammatory effects in a lipopolysaccharide-stimulated inflammatory model of intestinal epithelial Caco-2 cells co-cultured with RAW 264.7 macrophage cells [54]. However, the effects observed for these two compounds were less than the effects observed following treatment with SQE. Similarly, in the present study, *p*-coumaric acid and tricin suppressed self-renewal capacity, induced cell differentiation, and down-regulated the expression of various stem cell markers, less effectively than SQE. Furthermore, the combined treatment of *p*-coumaric acid and tricin had less effect on the suppression of sphere formation and stem cell marker expression, thereby confirming that other components of SQE are necessary for mediating the anti-CSC effects of SQE. These observations are consistent with those of previous studies where additive or synergistic effects of combined nutrients have been reported [59,60]. For example, the antioxidant activities of lycopene and other carotenoids from tomatoes have been found to be synergistic in protecting liposomes from oxidation compared with each carotenoid individually [61]. For the additive and synergistic antioxidant and anti-cancer activities of phytochemicals found in fruits and vegetables, these have been attributed to the complex mixture of these phytochemicals in whole foods [62]. Correspondingly, SQE, which includes a combination of polyphenols, has the potential to mediate additive anti-CSC effects in colon cancer.

## 3. Experimental Section

### 3.1. Preparation of SQE

SQE was prepared as previously described [54]. Briefly, harvested *Sasa quelpaertensis* Nakai bamboo leaves (1 kg) from Mt. Halla on Jeju Island in South Korea were cleaned and dried at 60 °C. The dried leaves were then extracted with 70% ethanol at room temperature. After 48 h, the SQE was filtered and concentrated using a rotary evaporator under reduced pressure, then was freeze-dried and ground into a powder. This SQE was stored at −20 °C until needed. The two major compounds in SQE are *p-*coumaric acid and tricin, and the concentrations of these compounds were determined using high performance liquid chromatography (HPLC) 2695 Alliance System (Waters Corp., Milford, MA, USA). The concentrations of *p-*coumaric acid and tricin were 1.13 and 0.82 mg/g, respectively [54].

### 3.2. Cell Culture

The human colon cancer cell lines, HT29 and HCT116, were purchased from American Type Culture Collection (ATCC, Rockville, MD, USA) and were cultured in McCoy’s 5A medium (Welgene, Daegu, Korea) supplemented with 10% fetal bovine serum (FBS) (Hyclone, Logan, UT, USA) and 1% penicillin-streptomycin (100 U/mL and 100 μg/mL, respectively) (Invitrogen, Carlsbad, CA, USA). The cells were maintained at 37 °C in a humidified 5% CO_2_ environment.

### 3.3. Isolation of CD133^+^CD44^+^ CSCs Using a Fluorescence-Activated Cell Sorting (FACS) System

Expression of the CSC markers, CD133 and CD44, were detected using flow cytometry. Briefly, HT29 and HCT116 cells were harvested with 0.05% trypsin and were washed with phosphate buffered saline (PBS). An Alexa Fluor 488 conjugated CD44 monoclonal antibody (Cell Signaling, Danvers, MA, USA) and a CD133 monoclonal antibody (Miltenyi Biotec, Bergisch Gladbach, Germany) were incubated with cells in the dark at 4 °C. After 30 min, the cells were washed and analyzed by a FACS DiVa system (BD, San Jose, CA, USA). Twenty percent of the cells with the highest fluorescence levels and the lowest fluorescence levels were selected as positive and negative controls, respectively.

### 3.4. Clonogenic Assays

To evaluate the self-renewal characteristics of the isolated colon CSCs, clonogenic assays were performed as previously described [14]. Briefly, CD133^+^CD44^+^ HT29 and HCT116 cells were plated in 6-well plates (300 cells/well) and then were treated with SQE (0, 100, 200, or 300 µg/mL), or comparable doses of *p-*coumaric acid and tricin. After 8 days, the colonies were fixed with 0.9% NaCl and were stained with crystal violet (Sigma Aldrich, St. Louis, MO, USA). At least 50 stained colonies were counted. Plating efficiency (PE) was calculated as follows: PE = (number of colonies/number of seeded cells) × 100% [14].

### 3.5. Sphere Formation Assays

Another assay to determine the self-renewal capacity of colon CSCs, sphere formation assay was performed as previously described [14]. Briefly, 6-well plates were coated with a 10% stock solution of poly-(2-hydroxyethyl methacrylate) (polyHEMA; Sigma Aldrich). Sphere medium was then added to each well [1:1 DMEM/F-12 medium (Welgene)] that was supplemented with 2% B27 (Invitrogen), 20 ng/mL human epidermal growth factor (EGF, Pepro Tech, London, UK), and 40 ng/mL basic fibroblast growth factor (bFGF, Pepro Tech). CD133^+^CD44^+^ HT29 and HCT116 cells (8 × 10^4^ cells/well) were treated with SQE (0, 100, 200, or 300 μg/mL), *p*-coumaric acid, and tricin. After 8 days, the number of spheres containing more than 50 cells were counted and photographed.

### 3.6. Antibodies and Western Blot Assays

Western blot assays were performed as previously described [53]. Briefly, cells and tumor tissues were lysed in RIPA buffer. Nuclear extracts was obtained using hypotonic and hypertonic buffer. These lysates and nuclear extracts were then separated by sulfate-polyacylamide gel electrophoresis (SDS-PAGE) and transferred onto polyvinylidene fluoride (PVDF) membranes (Millipore, Billerica, MA, USA). The membranes were incubated with 5% non-fat dried milk, and then were incubated overnight at 4 °C with primary antibodies raised against cytokeratin 20 (CK20, Abcam, Cambridge, MA, USA), DLK1, Sox-2, CD133 (Millipore), Notch1, HIF-1α (Novus Biologicals, Littleton, CO, USA), β-catenin, VEGF (Santa Cruz Biotechnology, Santa Cruz, CA, USA), CD44, Lamin A/C, or phospho GSK3β (Cell Signaling Technology, Danvers, MA, USA). The membranes were subsequently incubated with the appropriate secondary horseradish peroxidase IgGs, and bound antibodies were detected using enhanced chemiluminescence reagents (Animal Genetics Inc., Suwon, Kyonggi-do, Korea). Detection of α-tubulin (Sigma Aldrich) was used as a loading control.

### 3.7. Real-Time Quantitative PCR

Total cellular RNA was extracted using Trizol reagent (Invitrogen), and cDNA was synthesized using a RevertAid First Strand cDNA Synthesis Kit (Thermo Fisher Scientific, Waltham, MA, USA). PCR amplification was performed using Taq polymerase (TAKARA, Tokyo, Japan). Real-time PCR was performed using a Rotor-Gene Q instrument (Qiagen, Austin, TX, USA). Samples were combined with a Power SYBR Green PCR Master Mix (Qiagen, Hilden, Germany) and were subjected to the following conditions: initiation at 95 °C for 5 min, denaturation at 95 °C for 5 s, annealing at 60 °C for 10 s, and extension at 72 °C for 10 s. All data were normalized to the expression levels detected for β-actin and were analyzed by the CT method. The primers used included as follows: 5'-TGG ATG CAG AAC TTG ACA ACG T-3' (forward) and 5'-ATA CCT GCT ACG ACA GTC GTG GT-3' (reverse) for human CD133; 5'-GAG GCG TGG CAG ACT ATG C-3' (forward) and 5'-CTT GTA CTC CGT CAG CGT GA-3' (reverse) for human Notch1; 5'-AGCA CCC ATG GCA GAA GG-3' (forward) and 5'-CTC GAT TGG ATG GCA GTA CT-3' (reverse) for human VEGF; 5'-CCA ATG CCT TTG ATG GAC C-3' (forward) and 5'-TCT GTC TGT GCT GTC GGT GAT-3' (reverse) for human CD44; 5'-ATT GGC AAT GAG CGG TTC-3' (forward) and 5'-GGA TGC CAC AGG ACT CCA T-3' (reverse) for β-actin.

### 3.8. In Vivo Tumor Xenograft Model

Five-week-old male BALB/c nude mice (weight: 20–22 g) were purchased (Central Lab, Animal Inc., Seoul, Korea) and maintained under pathogen-free conditions (Ewha Laboratory Animal Genomic Center, Ewha Womans University, Seoul, Korea). Body weight (b.w.) and food intake were recorded twice a week.

To examine tumorigenicity of CD133^+^CD44^+^ double stained HT29 cells [1 × 104 cells (*n* = 4) and 1 × 105 cells (*n* = 6)] suspended in growth factor-reduced matrigel (BD Bioscience Laboratory, Bedford, MA, USA) were subcutaneously injected in the right flank of the BALB/c nude mice. These mice were sacrificed three weeks after injection and the tumorigenicity was examined.

To examine the effect of SQE on tumorigenicity, mice were randomly allocated into two groups (14 animals per group), (1) tumor control group (Ctrl) that received a standard diet and (2) SQE group (SQE) that received an oral supplement of 300 mg/kg b.w. SQE in distilled water. The latter group received SQE supplements five days a week for nine weeks. On day 14 of the SQE supplementation regimen, the mice were subcutaneously injected with 1 × 10^4^ CD133^+^CD44^+^ double-stained HT29 cells suspended in growth factor-reduced matrigel (BD Bioscience Laboratory, Bedford, MA, USA) into the right flank region. Tumor volume was measured twice a week using a digital caliper and was calculated as follows: (volume = length (mm) × width^2^·(mm^2^)/2). Six weeks after the injection of tumor cells, the mice were administered pimonidazole (60 mg/kg b.w.) 1 h prior to sacrifice. Upon resection of each tumors, the size and weight were measured, and then the tumors were immediately immersed in 4% formaldehyde or were frozen. Animal care and all experimental protocols were approved by the Animal Care and Use Committee of Ewha Womans University (IACUC approval No.: 2014-01-006).

### 3.9. Statistical Analysis

Statistical analyses were performed using GraphPad PRISM software (GraphPad Software, San Diego, CA, USA). The data are presented as the mean ± standard error of the mean (SEM). One-way analysis of variance (ANOVA) followed by Tukey’s post hoc test was used to analyze the five groups that were included in the *in vitro* study, while Student’s *t*-test was used to estimate differences between two groups included in the *in vivo* study. A *p*-value less than 0.05 was considered statistically significant.

## 4. Conclusions

In summary, SQE treatment inhibited colon CSCs both *in vitro* and *in vivo* by inducing cell differentiation, by suppressing tumorigenesis and the expression of CSC markers such as CD133, CD44, DLK1, Notch1, and Sox-2, and by regulating components of the Wnt/β-catenin and hypoxia related HIF-1α signaling pathways. These findings suggest that SQE may be a highly effective therapeutic strategy for controlling the growth of human malignant colon cancer cells.

## Abbreviations

ALDH1: Aldehyde dehydrogenase 1
bFGF: Basic fibroblast growth factor
CSC: Cancer stem cell
CK20: Cytokeratin 20
DLK1: Delta like 1 homologue
DMBA: 7,12-Dimethylbenz[α]anthracene
DSS: Dextran sulfate sodium
EGF: Epidermal growth factor
EPA: Eicosapentaenoic acid
FACS: Flow-activated cell sorting
GSK3β: Glycogen synthase kinase-3β
HEMA: Hydroxyethyl methacylate
HIF-1: Hypoxia-inducible factor-1
HPLC: High performance liquid chromatography
mTOR: Mammalian target of rapamycin
Notch1: Notch homolog 1, translocation-associated (Drosophila)
PBS: Phosphate buffered saline
P.C: *P*-coumaric acid
PE: Plating efficiency
PI3K: Phosphorylation of phosphoinositide-3-kinase
PVDF: Polyvinylidene fluoride
Sox-2: SRY-related HMG-box-2
SQE: *Sasa quelpaertensis* leaf extract
T: Tricin
TCF/LEF: T cell factor/lymphoid enhancer factor
VEGF: Vascular endothelial growth factor
